# Novel Polymeric Nanomaterial Based on Poly(Hydroxyethyl Methacrylate-Methacryloylamidophenylalanine) for Hypertension Treatment: Properties and Drug Release Characteristics

**DOI:** 10.3390/polym14225038

**Published:** 2022-11-21

**Authors:** Fevzi Bardakci, Kevser Kusat, Mohd Adnan, Riadh Badraoui, Mohammad Jahoor Alam, Mousa M. Alreshidi, Arif Jamal Siddiqui, Manojkumar Sachidanandan, Sinan Akgöl

**Affiliations:** 1 Department of Biology, College of Science, University of Hail, Hail P.O. Box 2440, Saudi Arabia; 2 Molecular Diagnostics and Personalized Therapeutics Unit, University of Hail, Hail P.O. Box 2440, Saudi Arabia; 3 Department of Chemistry, Faculty of Science, Dokuz Eylül University, Izmir 35390, Turkey; 4 Section of Histology-Cytology, Medicine Faculty of Tunis, University of Tunis El Manar, Tunis 1007, Tunisia; 5 Department of Oral Radiology, College of Dentistry, University of Hail, Hail P.O. Box 2440, Saudi Arabia; 6 Department of Biochemistry, Faculty of Science, Ege University, Izmir 35040, Turkey; 7 Nanotechnology Research and Application Center, Sabanci University, Istanbul 34956, Turkey

**Keywords:** polymeric nanomaterials, nanopolymer, drug release, amlodipine, hypertension

## Abstract

In this study, a novel polymeric nanomaterial was synthesized and characterized, and it its potential usability in hypertension treatment was demonstrated. For these purposes, a poly(hydroxyethyl methacrylate-methacryloylamidophenylalanine)-based polymeric nanomaterial (p(HEMPA)) was synthesized using a mini-emulsion polymerization technique. The nanomaterials were characterized using scanning electron microscopy (SEM), Fourier transform infrared spectroscopy (FTIR), and zeta size analysis. The synthesized p(HEMPA) nanomaterial had a diameter of about 113 nm. Amlodipine-binding studies were optimized by changing the reaction conditions. Under optimum conditions, amlodipine’s maximum adsorption value (Qmax) of the p(HEMPA) nanopolymer was found to be 145.8 mg/g. In vitro controlled drug release rates of amlodipine, bound to the nanopolymer at the optimum conditions, were studied with the dialysis method in a simulated gastrointestinal system with pH values of 1.2, 6.8 and 7.4. It was found that 99.5% of amlodipine loaded on the nanomaterial was released at pH 7.4 and 72 h. Even after 72 h, no difference was observed in the release of AML. It can be said that the synthesized nanomaterial is suitable for oral amlodipine release. In conclusion, the synthesized nanomaterial was studied for the first time in the literature as a drug delivery system for use in the treatment of hypertension. In addition, AML–p(HEMPA) nanomaterials may enable less frequent drug uptake, have higher bioavailability, and allow for prolonged release with minimal side effects.

## 1. Introduction

Cardiovascular diseases are one of the world’s most important causes of death [1]. Hypertension is a prominent risk factor for cardiovascular, cerebrovascular, and renal complications. Therefore, hypertensive patients need to take several drugs daily to regulate their blood pressure. Moreover, hypertension side effects may increase if a patient forgets to take the drugs. It is undesirable for blood pressure to drop too slowly or rapidly. Therefore, stroke paraplegia, death, etc., may cause the hypoperfusion of the central nervous system, which can lead to [2,3] hypertension with blood pressure above the desired values, which is described as a blood pressure ≥140/90 mmHg [4]. Angiotensin-converting enzyme inhibitors (ACEIs), ß-blockers, and calcium channel blockers (CCBs), used as first-line anti-hypertensive drugs, have been shown to exert potent and stable anti-hypertensive effects on hypertension. ACEIs and ß-blockers block the renin angiotensin system. CCBs, on the other hand, help increase blood pressure by increasing the flow of Ca^+2^ into cells [1,5,6]. Anti-hypertensive treatment based on dihydropyridine calcium channel blockers with a high vascular selectivity, which is currently among the used pharmacological therapies for the clinical management of hypertension, has been shown to be effective, safe, and well-tolerated in reducing the blood pressure levels and organ damage caused by hypertension [7]. CCBs are classified according to their actions: (1) short-acting agents (nicardipine, diltiazem, and verapamil), (2) long-term released agents that are time set and taken once a day, and (3) natural agents that have long-acting effects such as amlodipine [8]. 

Amlodipine besylate (AML) is a long-acting dihydropyridine calcium antagonist. It inhibits the transmembrane flow of Ca^+2^ ions to vascular smooth and cardiac muscles. Suggested oral dosages of AML are from 2.5 to 10 mg/day. AML is almost completely absorbed immediately after oral administration, after which absorption is slowed. Its low water solubility and low rate of permeability through the gastrointestinal tract limit the access of AML to its therapeutic targets, such as the heart and cardiac smooth muscle. Therefore, its bioavailability is relatively low (60–65%) [9,10,11,12]. 

AML is used in managing hypertension, chronic stable angina pectoris, and Prinzmetal’s variant angina [13]. The therapeutic concentration level for AML in plasma is between 1 and 25 ng/mL. It is highest in plasma at 6 to 9 h after oral administration [11,14]. Studies have shown that AML also has a significant antioxidant activity and plays a vital role in apoptosis [15]. Therefore, the therapeutic efficacy of AML is not limited to the treatment of cardiovascular diseases. AML is also used in the treatment of different diseases (cerebrovascular stroke, neurodegeneration, cancer, etc.) alone or in combination with other therapeutics [16].

In recent years, there has been an increasing interest in drug release from nanomaterials as an important approach to treating various diseases. The combination of multiple functional modules into a single nanocarrier can increase the intracellular delivery of many drugs, which might enhance their therapeutic efficacy [1]. In recent years, many studies on the oral release of macromolecule therapeutics from nanomaterials have been published. In some of these studies, applications were carried out with absorption enhancers, while in others, modifications of the drug molecule were carried out. Nanopolymer-based drug release systems can prevent the structural degradation of drugs in the gastrointestinal tract and increase contact with the mucosal membrane. In this way, the bioavailability of a drug may increase. Nevertheless, the behavior of nanopolymers depends on different physicochemical properties (size, surface charge, hydrophilicity, etc.) [17,18,19,20,21,22,23].

The main motivation for this study was to explore the potential of an improved oral controlled release system without side effects, increasing the bioavailability and decreasing the frequency of intake of AML. For this purpose, we constructed a model nanopolymer, comprising a monomer for HEMA and a comonomer for MPA, which could interact with AML through secondary interactions, such as hydrogen bridge bonds, hydrophobic interactions, and electrostatic interactions. The obtained nanopolymer was characterized in morphology, particle size, and zeta potential. Optimum adsorption conditions (pH, time, initial concentration, and temperature) were determined for the maximum adsorption capacity of AML onto the synthesized nanopolymers. In vitro drug release studies were performed under conditions that mimicked gastrointestinal conditions. Therefore, this study was able to assess the controlled release of AML from a p(HEMPA) nanopolymer for hypertension treatment.

## 2. Materials and Methods

### 2.1. Materials

L-phenylalanine methyl ester hydrochloride, methacryloyl chloride, 2-hydroxyethyl methacrylate, ethylene dimethacrylate, poly(vinyl alcohol) (fully hydrolyzed), ammonium persulfate (APS) (ACS reagent, ≥98.0%), sodium dodecyl sulfate (ACS reagent, ≥99.0%), sodium bisulfite, sodium carbonate, sodium nitrite, potassium carbonate, and amlodipine besylate were obtained from Sigma-Aldrich^®^ (Munich, Germany).

### 2.2. Methods

#### 2.2.1. Preparation of p(HEMPA) Nanomaterials

##### Synthesis of MPA 

N-methacryloyl-(L)-phenylalanine methyl ester (MPA) was synthesized following the methods described in a previous study [24]. For MPA synthesis, 5 g (0.02 mol) of (L)-phenylalanine methyl ester ((C_6_H_5_CH_2_CH(NH_2_)COOCH_3_)) and 0.2 g (2.9 × 10^−3^ mol) of sodium nitrite were dissolved in a 30 mL potassium carbonate solution (5%, *w*/*v*). The mixture was put into a round-bottomed three-neck flask equipped with a dropping funnel. The reaction medium was cooled to 0 °C. Methacryloyl chloride (C_4_H_5_ClO) (4.0 mL) was slowly poured into this solution under a nitrogen atmosphere, and this solution was then magnetically stirred at room temperature for 2 h. At the end of this chemical reaction period, the pH of the solution was adjusted to 7.0 with 0.5 M NaOH, and the solution was subsequently extracted with ethyl acetate. The MPA was allowed to crystallize in an ether-cyclohexane mixture on an evaporator apparatus.

##### Preparation of p(HEMPA) Nanopolymers

The polymerization process was carried out with a mini-emulsion polymerization technique. There are two liquid phases in this method. In the first liquid phase, 93.8 g (9.4 × 10^−4^ mol) of PVA as a stabilizer, 14.43 g (0.05 mol) of SDS as a surfactant, and 11.73 mg (1.4 × 10^−4^ mol) of sodium bicarbonate in an aqueous solution (5 mL) were used. In the second liquid phase, aqueous solutions of 50 mg (5 × 10^−7^ mol) of PVA and 50 mg (1.7 × 10^−4^ mol) of SDS were used. The monomer phase was prepared with HEMA (0.65 μmol) and EGDMA (6.5 μmol). The monomer phase prepared in this way was added to the first liquid phase, and the mixture was stirred to obtain a mini-emulsion. The prepared MPA monomer was added to the mini-emulsion and mixed. The mini-emulsion was slowly added to the second liquid phase and mixed. The mixture was transferred to a glass polymerization reactor and passed through nitrogen gas. Finally, 45 mL of ammonium persulfate (0.44 mg/mL) was added to the mixture. The mixture was polymerized at 40 °C for 24 h. The resulting p(HEMPA) nanopolymers were washed with purified water and ethanol several times.

#### 2.2.2. Characterization Studies

The FTIR spectrum of synthesized p(HEMPA) nanopolymer was obtained using FTIR spectrophotometer (PerkinElmer, Waltham, MA, USA). Nanopolymers (2 mg) were dried at 45 °C for 48 h and made ready for subsequent analysis. The FTIR-ATR spectra of the samples were analyzed with a wave count range of 4000–600 cm^−1^.

The particle size of the synthesized p(HEMPA) nanopolymer was analyzed with a Nano Zetasizer (NanoS, Malvern Instruments, London, UK).

To investigate the surface properties and particle size of p(HEMPA) nanopolymer, nanopolymers were first dried at 45°C for 48 hours and then coated with gold to increase the conductivity of the polymer, and the images were taken with the SEM device (ThermoFisher Scientific, Waltham, MA, USA). Elemental analysis was performed using Bruker model X flash 6/10 (Bruker, Rheinstetten, Germany).

The surface area of the nanopolymers was calculated as in previous studies [25].

#### 2.2.3. Binding Studies of Amlodipine

Binding studies were performed in triplicate for each parameter.

#### 2.2.4. Effect of Nanopolymer: Drug Ratio

The adsorption spectra of the samples were analyzed in the range of 200–400 nm [10,26]. The efficiency of the nanopolymer:amlodipine (AML) ratio was determined by calculating the amount of the unbound drug. The free AML concentration in the solution was measured at 364 nm [27].

The adsorption capacity was calculated as in previous studies in accordance with the following Equation [28]:

Q = [(C_0_ − C)V]/m

Q: AML amount bound to 1 g of nanopolymer (mg/g);
C_0_: AML initial concentration;
C: AML concentration in the solution (mg/mL);
V: solution volume (mL);
M: nanopolymer mass (g).

#### 2.2.5. Optimization of AML Release Conditions

The effects of AML concentration, time, and temperature parameters on the AML release of the p(HEMPA) nanopolymer were investigated. The effect of the medium pH on the AML release from the p(HEMPA) nanopolymer was studied using buffer solutions (pH: 1–8). The p(HEMPA) nanopolymer was reacted with 1.0 mL of a buffer solution under optimum reaction conditions, and the AML release was spectrophotometrically determined at 364 nm. To examine the effect of temperature on AML release, the p(HEMPA) nanopolymer was mixed with a 1 mL phosphate buffer solution (100 mM and pH 7.4), and AML release was monitored at different temperatures (4 °C, 25 °C, and 37 °C). All experiments were performed in 3 repetitions.

#### 2.2.6. In Vitro AML Release Studies

Studies of AML release from the AML loaded-p(HEMPA) nanopolymers were performed according to the method described by Chen et al. (2009) [29]. The in vitro release of AML from the AML-loaded p(HEMPA) nanopolymer was examined in gradient pH media. For this purpose, the medium pH was changed by simulating the gastrointestinal fluid (SGF) (pH 1.2) and intestinal fluid (SIF) (pH 6.8 and pH 7.4) pHs. The amount of AML released from the AML-loaded nanopolymers was determined by dialysis at 37 °C (dialysis bag: MWCO 12–14 kDa). In summary, the medium temperature in the water bath was kept at 37 ± 0.5 °C with magnetic stirring. At specified time intervals, samples were taken and replaced with an equal volume of fresh medium. The samples were measured with a UV spectrophotometer at a wavelength of 364 nm [30]. All experiments were performed in 3 repetitions.

Various mathematical models were used to analyze the mechanism of drug release from the AML-loaded nanopolymer system. Data were fitted into several kinetic models. These kinetic models were zero order, first order, Higuchi equation, Hixson–Crowell equation, and Korsmeyer Peppas [31,32]. The regression analysis was performed on all kinetic models, and the most suitable model was selected according to the calculated correlation coefficients (R^2^).

## 3. Results and Discussion

### 3.1. Characterization of p(HEMPA) Nanopolymer

**Figure 1:**
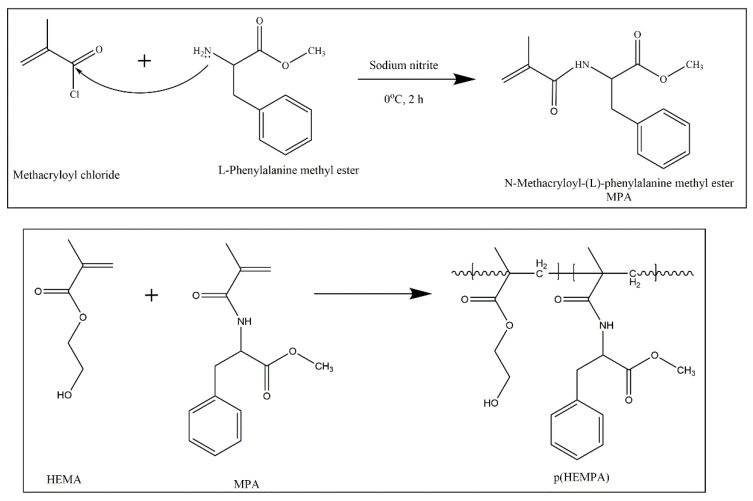
Hypothetical structure of p(HEMPA) nanopolymer synthesized using the mini-emulsion polymerization technique.

**Figure 2:**
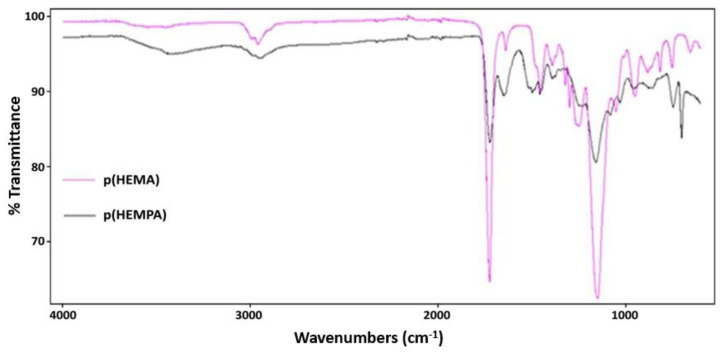
FTIR spectra of p(HEMPA) nanopolymer synthesized using the mini-emulsion polymerization technique.

The molecular structure and FTIR spectra of the studied p(HEMPA) nanopolymer are shown in Figure 1 and Figure 2, respectively. The peak at 700.66 cm^−1^ in the p(HEMPA) spectrum was caused by the aromatic properties of p(HEMPA). Intensive peaks at 1029.09 cm^−1^, 1079.8081 cm^−1^, and 1238.80 cm^−1^ correspond to the C-N stretching of p(HEMPA). An intensive peak at 1344.31 cm^−1^ corresponds to the C-N stretching of aromatic amine. The absorption peak at 1650.92 cm^−1^ corresponds to the characteristic N-H binding of p(HEMPA). The intensive peak at 1721.67 cm^−1^ corresponds to the C=O stretching of p(HEMA). The stretching vibration of the –OH group of p(HEMPA) was observed around 3424.92 cm^−1^. Accordingly, it was observed that the MPA monomer successfully formed a copolymer with the HEMA monomer (Figure 1).

**Figure 3:**
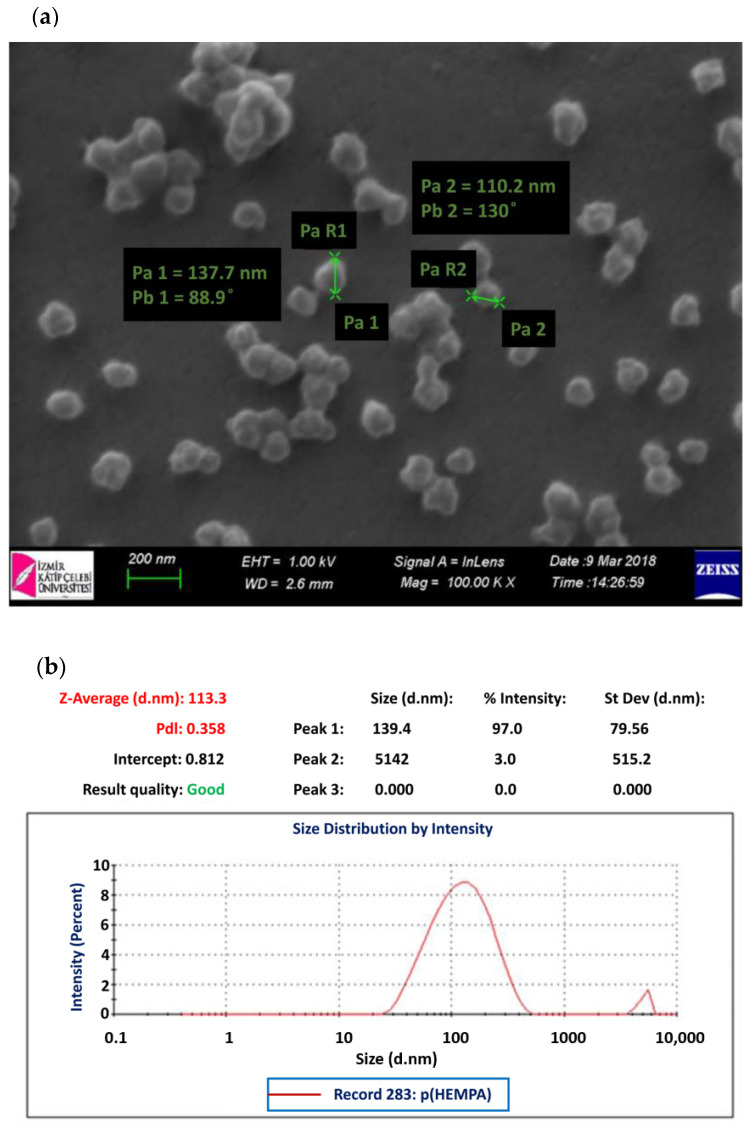
HEMPA nanopolymer synthesized using the mini-emulsion polymerization technique: (**a**) SEM micrographs; (**b**) zeta sizer analysis.

**Figure 4:**
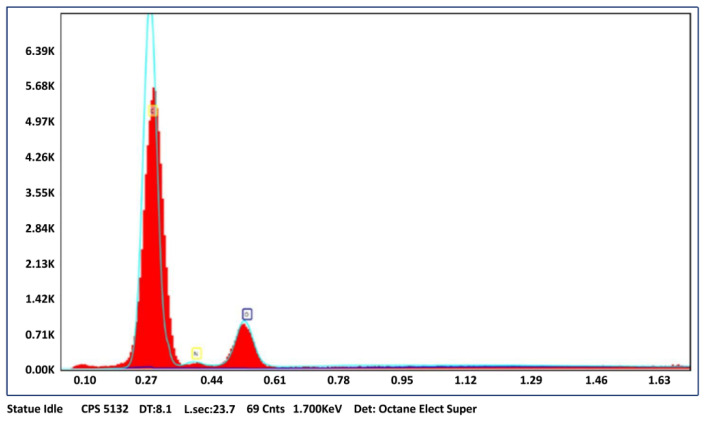
EDS spectra of HEMPA nanopolymer synthesized using the mini-emulsion polymerization technique.

SEM micrographs of p(HEMPA) are presented in Figure 3a. The spherical character of the nanopolymers was demonstrated by SEM. Although the nanopolymer was dispersed in a medium-polar solvent such as ethanol, nanopolymer particles were integrated, as seen in the SEM images. This was due to the hydrophobic interactions between the nanopolymer particles as a result of the hydrophobic structure of the MPA monomer. According to the zeta size analysis, the average size of the p(HEMPA) nanopolymers synthesized for use in AML release studies was 113.30 nm (Figure 3b). The EDS spectra of p(HEMPA) nanopolymers are shown in Figure 4, in which the peak intensities of N can be identified, and the percentage composition was found to be 4.8%. The presence of nitrogen in the EDS results is another indication that the functional MPA monomer successfully formed a copolymer with the HEMA monomer.

### 3.2. AML Binding to the Nanopolymer

**Figure 5:**
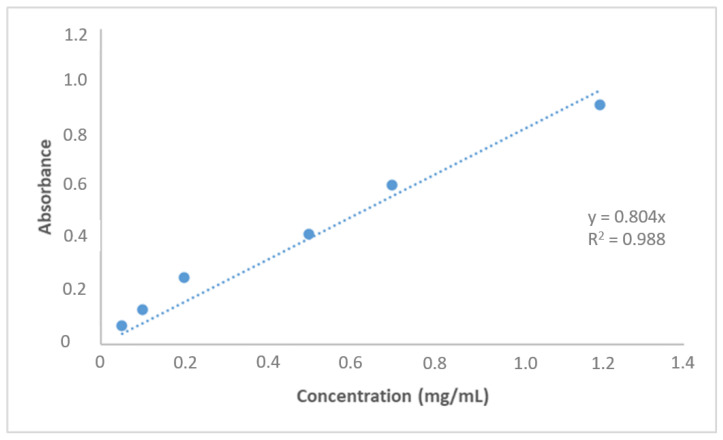
The standard curve for the detection of amlodipine.

A standard curve for the detection of amlodipine is shown in Figure 5. The effect of pH on AML’s ability to bind to nanopolymers was investigated in this study. The optimum pH value was found to be pH 7.4 in the phosphate buffer. Therefore, other binding experiments were carried out with the pH 7.4 phosphate buffer. The solubility of AML in water and ionization at physiological pH (pKa 8.7) were found to be better than those of other dihydropyridine group molecules [33] because the maximum adsorption was theoretically expected to increase as it approached the pKa value. The dominant forces were non-covalent interactions between the AML and nanopolymer.

**Figure 6:**
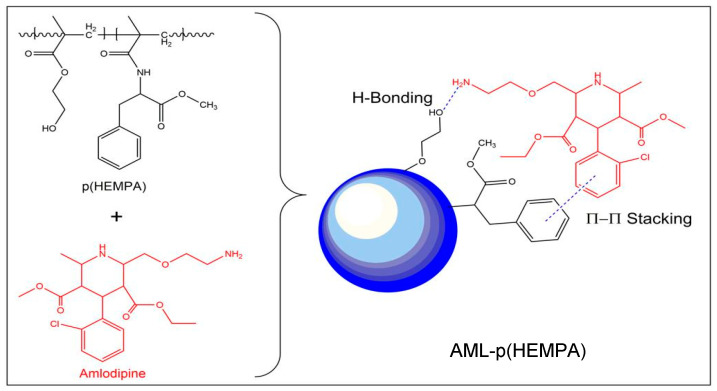
Hypothetic molecular structure of amlodipine loaded AML–p(HEMPA) nanopolymers.

It is thought that the dominant forces between the MPA used as the functional monomer and AML were hydrophobic interactions and hydrogen bridge bonds that formed between the amlodipine molecule’s -CH_3_ group on the pyridine ring and the hydroxyl group of the HEMA monomer (Figure 6). A decrease in adsorption capacity and the pKa value of AML was attributed to the deprotonation of the -CH_3_ group on the pyridine ring. Binding experiments were carried out between 4 and 37 °C. We found that the optimum temperature was 37 °C. Above this temperature, there was a rapid decrease in the binding amount of AML. It is known that as temperature increases, the van der Waals forces also increase. According to the theory developed for hydrophobic substances dissolved in water, entropy is the driving force in the process of binding to hydrophobic adsorbents [ΔG = (ΔH − TΔS)], where ΔH can be positive or negative and the control of ΔG is provided by the positive entropy change. For this reason, the entropy increased with temperature. The increased binding capacity with increasing temperature indicated the binding between hydrophobic phenylalanine and AML. Furthermore, since physical adsorption is an exothermic process, it is expected to be higher at lower temperatures but to decrease with increasing temperature.

**Figure 7:**
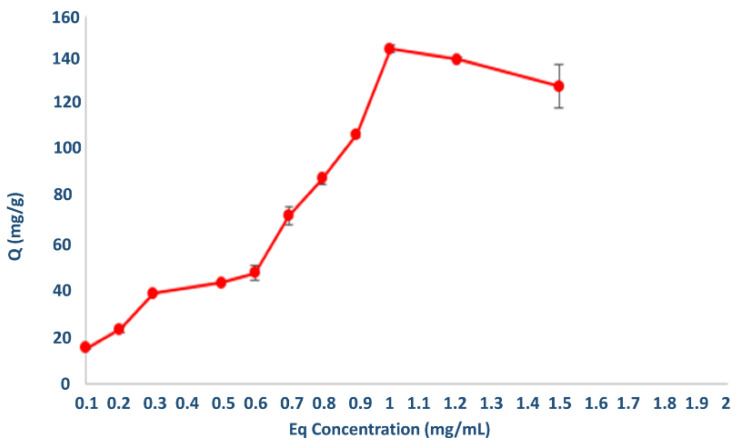
Effect of initial AML concentration on the adsorption capacity of p(HEMPA) (100 mM phosphate buffer pH 7.4, 60 min, and 25 °C).

As shown in Figure 7, the amount of AML bound by nanopolymers increased with the concentration of AML in the solution. The concentration difference (ΔC) was the driving force of the AML binding to the nanopolymers. The greater this concentration difference, the greater the amount of AML binding. As expected, with the increase in the driving force, the binding capacity was observed to increase. Nanopolymer reached their maximum adsorption capacity (145.8 mg/g) at a 1 mg/mL AML concentration, and the adsorption capacity did not significantly increase above this value. To compare the performance of the p(HEMPA) nanopolymer with previous studies in the literature: Kapoor et al. optimized amlodipine nanostructured lipid carriers (AMNLCopt) showed a low particle size (123.8 nm), enhanced transdermal flux (58.33 μg/cm^2^/h), and higher entrapment efficiency (88.11%) [27]. In another study, Uthaman and Koland found that the maximum drug incorporation or entrapment efficiency in micellar dispersions was 91.82% in amlodipine besylate-loaded polymeric micelles [34].

### 3.3. In Vitro AML Release Studies and Kinetics

**Figure 8:**
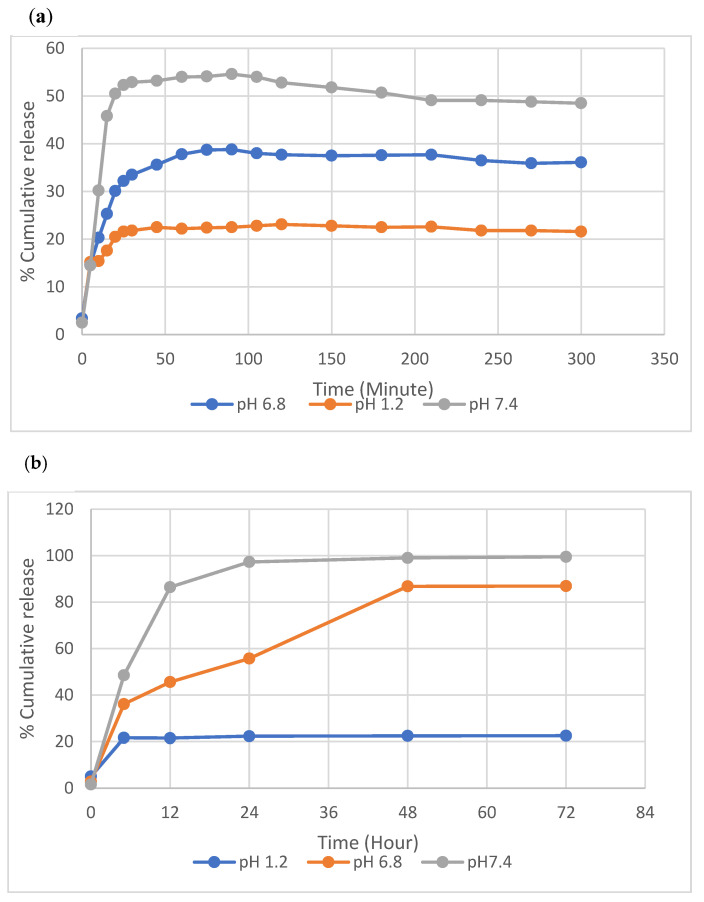
Cumulative release of AML at the different time points (minute (**a**) and hour (**b**)) in vitro from AML–p(HEMPA) nanopolymers (loaded AML amount: 1 mg/mL nanopolymer; T: 37 °C).

The release of AML was evaluated with AML-loaded-nanopolymers in SGF without enzymes with the dialysis method. Figure 8a,b shows the total release of AML from the p(HEMPA) nanopolymers at different time intervals in vitro.

The interactions between AML and p(HEMPA) nanopolymers are thought to comprise hydrophobic interactions and hydrogen bridge bonds. In SGF (pH 1.2), the AML–p(HEMPA) drug conjugate showed AML release rates of 21.5%, 22.3%, 22.4%, and 22.5% in 12 h, 24 h, 48 h, and 72 h, respectively. Even after 72 h, no difference was observed in the release of AML. At pH 1.2, the release of AML was slow and stable as a result of hydrogen bridge bonds between the protonated form of the –NH group in the pyridine ring of the AML and the –OH group of the p(HEMPA) nanopolymer. In addition, there were hydrophobic interactions between functional MPA monomer and the nitrobenzene and CH_3_ groups of AML. These results also indicate that the AML-loaded nanopolymer remained stable in a gastric fluid environment, whereas at pH 6.8, the AML–p(HEMPA) drug conjugate showed 45.6%, 55.8%, 86.8%, and 89.9% release rates of AML in 12 h, 24 h, 48 h, and 72 h, respectively. The reason for the rapid release of AML at pH 6.8 is thought to be the decrease in the interaction between the nanopolymer and drug as a result of AML deprotonation. The release amount of AML at an acidic pH was lower than the release amount at a neutral pH; this indicates that the release of AML from the AML–p(HEMPA) nanopolymer will be suppressed in the gastrointestinal tract and that AML release can be promoted when the nanopolymers enter the small intestine. At the same time, the AML–p(HEMPA) drug conjugate at pH 7.4 showed 86.4%, 97.3%, 99.02%, and 99.5% release rates of AML at 12 h, 24 h, 48 h, and 72 h, respectively. Similar to our results, Mei et al. reported that in the highly acidic pH environment of the stomach, a network formed due to the electrostatic interactions of the oppositely charged polymers and resulted in stronger retention of the drug in the polymer [35]. Another study indicated that these anti-hypertensive-acting nanopolymers will release minimal drugs until they reach the next part of the gastrointestinal tract. Due to the high pH of the tract, nanopolymers are neutral, and the interaction between polymers decreases at higher pH values, thus causing drug release [36].

The drug release data were fitted to several kinetic models of AML release from the p(HEMPA) nanopolymer. The value of the correlation coefficient for release data at pH 7.4 was given by the Higuchi model, which was fitted best according to the value of the correlation coefficient (R^2^ = 0.9947) (Table 1). This model is based on the hypothesis that the initial drug concentration in a nanomaterial is higher than drug solubility. The Higuchi model is the relation between the cumulative percentage of released drugs and the square root of time. This model is useful for studying the release of water-soluble and poorly soluble drugs from a variety of matrices, including solids and semisolids [37].

Similarly, Kapoor et al. also found that the best fit model was the Higuchi model (0.981) for an amlodipine nano lipid carrier. The Peppas model showed an n value of 0.344, indicating that drug release was regulated through non-Fickian diffusion [27]. In another study, Khushbu et al. attempted to gain insight into the kinetics of amlodipine release from CH/ALG/GO nanocomposites at different pH values; the release profile was studied by using six kinetic models [38]. To explain the kinetic behavior in this study, the Korsmeyer–Peppas model was only applied to a part of the release curve where the aggregate drug release fraction was below 60%. That is why six kinetic models were used to study the release profile. According to the rule, the model with the highest correlation coefficient (R^2^) value was the best-fitting model. As can be seen from the results, the value of R^2^ was the highest for the Peppas–Sahlin equation in almost all pH environments and the Korsmeyer–Peppas equation at pHs of 7 and 7.4; therefore, these two models were best fit for APB release. The first order and zero-order equations did not follow the drug release. The relative contribution from the diffusion and relaxation process was evaluated with the Peppas–Sahlin equation. Data analysis showed that the Higuchi and Hixon-Crowell models did not follow release kinetics.

## 4. Conclusions

In conclusion, the current study was undertaken to design a new polymeric nanomaterial and to evaluate the controlled-release oral administration of AML. For this purpose, poly(hydroxyethyl methacrylate-methacryloylamidophenylalanine)-based polymeric nanomaterials (p(HEMPA)) were synthesized using a mini-emulsion polymerization technique. The synthesized p(HEMPA) nanomaterial is about 113 nm in diameter. Amlodipine-binding studies were optimized by changing the reaction conditions. Under optimum conditions, amlodipine’s maximum adsorption value (Qmax) of the p(HEMPA) nanopolymer was found to be 145.8 mg/g. In vitro controlled drug release studies of amlodipine, bound to the nanopolymer at the optimum conditions, were conducted in a simulated gastrointestinal system with pH values of 1.2, 6.8 and 7.4. It was found that 99.5% of amlodipine loaded on the nanomaterial was released at pH 7.4 and 72 h. Even after 72 h, no difference was observed in the release of AML. The drug release data were fitted to several kinetic models of AML release from the p(HEMPA) nanopolymer. The value of the correlation coefficient for release data in pH 7.4 was given by the Higuchi model, which was fitted best according to the value of the correlation coefficient (R^2^ = 0.9947). As a result, AML–p(HEMPA) nanomaterials may enable less frequent drug uptake, have higher bioavailability, and allow for prolonged release with minimal side effects. Evidently, it can be concluded that AML–p(HEMPA) nanopolymers may increase the oral bioavailability of hydrophobic agents, increase plasma half-life, and minimize the side effects associated with anti-hypertensive drugs by reducing their dosage requirements and intake frequency.

## Figures and Tables

**Table 1:** Correlation coefficient (R^2^) for various mathematical models.

Correlation Coefficient (R^2^)					
Sample	Zero Order	First Order	Higuchi	Korsmeyer–Peppas	Hixson–Crowell
p(HEMPA)/AML	0.9751	0.9736	0.9947	0.9660	0.9634

## Data Availability

Not applicable.
